# Substrate Based Ablation of Ventricular Tachycardia Through An Epicardial Approach

**Published:** 2009-11-01

**Authors:** Aman Makhija, Ajit Thachil, C Sridevi, B Hygriv Rao, S Jaishankar, Calambur Narasimhan

**Affiliations:** 1 Fellow Electrophysiology, CARE Hospital, Institute of Medical Sciences, Hyderabad, India.; 2 Consultant Electrophysiologist & Sr Cardiologist, CARE Hospital, Institute of Medical Sciences, Hyderabad, India.; 3 Director Electrophysiology & Pacing, CARE Hospital, Institute of Medical Sciences, Hyderabad, India.

**Keywords:** ventricular tachycardia, substrate based mapping, epicardial approach, electroanatomic mapping

## Abstract

Ventricular tachycardia (VT) occurring late after myocardial infarction is often due to reentry circuit in the peri-infarct zone. The circuit is usually located in the sub-endocardium, though subepicardial substrates are known. Activation mapping during VT to identify target regions for ablation can be difficult if VT is non inducible or poorly tolerated. In the latter, a substrate based approach of mapping during sinus rhythm in conjunction with pace mapping helps to define the reentry circuit and select target sites for ablation in majority of patients with hemodynamically unstable VT. Percutaneous epicardial catheter ablation has been attempted as an approach where ablation by a conventional endocardial access has been unsuccessful. We report a case of post myocardial infarction scar VT which could be successfully ablated with a substrate based approach from the epicardial aspect.

## Case report

A 62 year old male presented with recurrent ventricular tachycardia (VT). He had suffered an inferior wall myocardial infarction with resultant mild left ventricular (LV) dysfunction (LV ejection fraction 40%) and had an ICD implanted 3 years back. The patient was now hospitalised with recurrent appropriate ICD shocks. He was loaded upfront with intravenous amiodarone followed by oral amiodarone and beta blockers. However, VT continued to recur resulting in frequent ICD shocks despite maximal tolerated drug therapy.

**Figure 1:**
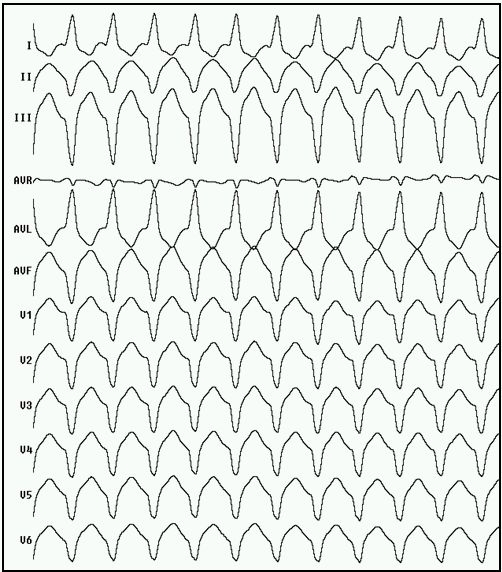
Ventricular tachycardia with LBBB, left superior axis morphology

Patient was taken up for radiofrequency ablation. An initial endocardial approach was used using electro-anatomic mapping system (EAM) (CARTO, Biosense Webster, Inc. Diamond Bar, CA, USA). The equipment used for recording was an EP-TRACER (Cardio Tek, Maastricht, Netherlands). An endocardial voltage map of the LV was created using a Navistar catheter under EAM guidance by a retrograde transfemoral approach. While mapping, a sustained monomorphic VT was induced with a LBBB, superior axis morphology, QS complexes in V1-V6; tachycardia cycle length (TCL) of 318 msec (Figure 1). The endocardial activation map from the earliest to the latest signal encompassed for only 40% of the duration of tachycardia cycle suggesting that most of the reentry VT circuit was located elsewhere, likely in an epicardial location. A decision to approach the pericardial space for mapping was made.

The pericardial space was exposed using a percutaneous subxiphoid approach. Pericardial puncture was performed with a 17-gauge epidural needle. The puncture was performed at the left lower edge of the xiphoid, at a 45 degree angle toward the left scapula. Two milliliters of contrast was injected via the needle to delineate the heart silhouette to confirm intra-pericardial location. A standard 8F sheath mounted over a guidewire was then introduced into the pericardium through a small incision. An externally irrigated RF catheter (Thermo cool Celsius, Cordis Webster, Inc. Diamond Bar, CA, USA) was then introduced through the sheath and carefully pushed into the pericardial space. The catheter was constantly irrigated with saline (1 mL/min flow rate). Programmed electrical stimulation (PES) from the epicardium failed to induce the VT. Following isoprenaline infusion VT could be induced on PES. However the VT was hemodynamically unstable and had to be promptly terminated.

**Figure 2:**
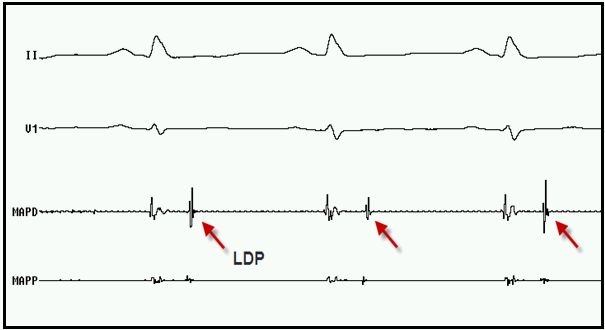
Electrogram demonstrating Epicardial mapping catheter (MAPD) recording late diastolic potentials (LDP) during sinus rhythm. From top to bottom, surface electrograms II and V1; intracardiac electrograms from distal (MAPD) and proximal (MAPP) epicardial ablation catheter.

**Figure 3:**
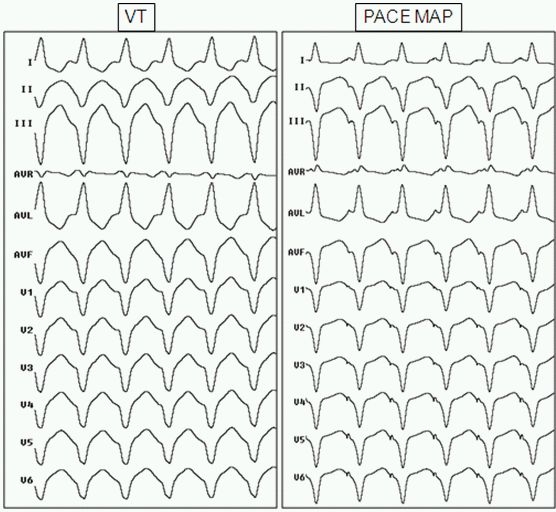
Ventricular Tachycardia and good 12/12 pace map match

**Figure 4:**
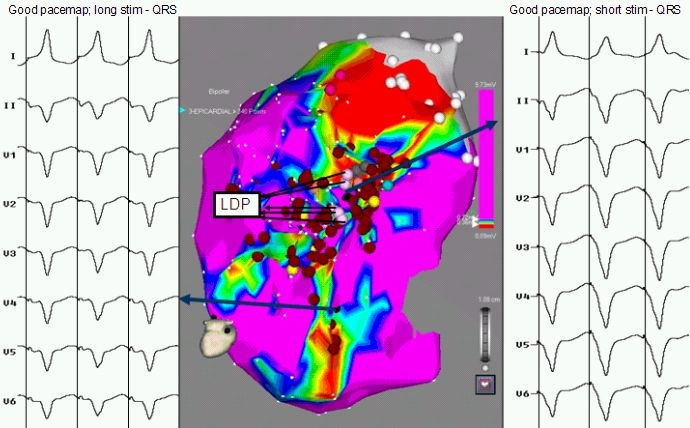
LV Epicardial voltage map. Inferior view recorded during sinus rhythm. Demonstrating a large postero-basal scar. Areas in purple display normal voltage (> 1.5 mV); areas in red display more dense scar (<  0.5 mV). Arrows depict two sites with late diastolic potentials, good pace mapping, short (S-QRS = 20 msec) and long (S-QRS = 70 msec) consistent with exit and entry sites in reentry circuit. Pink dots represent late diastolic potentials (LDP), red dots indicate site of ablation.

A substrate based voltage map of the LV epicardium in sinus rhythm was constructed which revealed presence of scar (defined by voltages < 0.5 mV) in posterobasal LV. Multiple areas of low voltage fractionated activity in the peri-scar area associated with late diastolic potentials (LDP) during sinus rhythm could be identified (Figure 2). These areas demonstrated reversal of activation sequence during non sustained VT with the latest potentials now preceding the QRS during tachycardia. Pace mapping from these areas gave a 11/12 match to the initial sustained VT (Figure 3); two sites with stimulus to QRS duration of 20 msec (exit site) and 70 msec (entry site) (Figure 4) during the pace mapping were delineated. Left coronary angiogram was done to exclude any major coronary artery close to the mapping catheter before ablation. Late Diastolic Potentials (LDP) in low voltage areas (which were clustered in postero-basal LV) (Figure 4) with good pace mapping were targeted with RF energy using 40W and 20 ml/min irrigation for 120 sec. Post RFA, VT was not inducible with aggressive stimulation protocol (extra stimuli/burst pacing) at baseline and on isoprenaline. Post procedure hydrocortisone 100 mg was injected into the pericardial space and also sintravenously to avoid any pericardial reaction. A pigtail catheter was left in the pericardial space for 24 hours to drain any residual pericardial fluid.

## Follow up

A review EP study was performed 24 hours later which did not induce any tachycardia on PES with isoprenaline. Oral amiodarone was stopped and the patient was discharged on beta blockers. Patient remained symptom free and at a follow up of three months, the ICD did not reveal  any new tachyarrhythmia by telemetry.

## Discussion

Catheter ablation has an increasingly important role in controlling ventricular tachycardia (VT) episodes in patients with structural heart disease who have implanted cardioverter defibrillators. Substrate for reentrant VT is often subendocardial and is accessible for catheter ablation by a transvenous approach. However, epicardial ablation is required for 10-30% of postinfarct VTs, and for more than 30% of VTs due to nonischemic cardiomyopathy [1]. Percutaneous access to the pericardial space has improved our ability to treat these patients with catheter ablation with minimal morbidity. A substrate based mapping and ablation of a hemodynamically unstable VT was successfully performed via an epicardial approach in our patient.

The substrate for postinfarction VT is well characterized. Surviving muscle bundles commonly located in the subendocardium but also in the mid myocardium and epicardium, traverse the borders and penetrate the deeper scar [2]. The location of surviving muscle bundles within the scar can be identi?ed by the detection of delayed low-voltage diastolic potentials, which occur "after completion of the surface QRS". During sinus rhythm (SR), these sites are activated from the borders of the endocardial scar, or potentially from the epicardial surface. Sites with the latest activation are often, although not invariably, located in the more central portions of the scar. During VT, some of these sites may be within the slow conduction channel, with local activation during later diastole preceding the onset of surface QRS [3]. A good pace map identical to the clinical VT helps to identify slow conduction channels participating in the tachycardia, rather than dead-end pathways or blind alleys [4]. Exit sites at the scar border defined by presence of late diastolic potentials, pace-maps with exact reproduction of the VT morphology and short stimulus-QRS intervals were targeted for ablation in our case.

Ciccasio et al [5] used sinus rhythm activation maps to detect the origin and characteristics of reentrant ventricular tachycardia in 11 postinfarction patients. The estimated isthmus, determined from sinus rhythm activation, overlapped the diastolic pathway, determined from tachycardia maps, with 83.8% sensitivity and 89.2% specificity. In another study, substrate ablation in patients with a previous myocardial infarction with ICD resulted in 65% reduction in the risk of receiving ICD therapy during a 2 years follow up [6].

## Conclusion

Percutaneous, pericardial instrumentation via subxiphiod approach permits characterization and imaging of the epicardial substrate in a fashion similar to that for the endocardium. A substrate based ablation reduces or eliminates the need for mapping during prolonged periods of tachycardia and extends the potential benefits of ablation to the large majority of VTs that are hemodynamically unstable.
